# Pd-Ceria/CNMs Composites as Catalysts for CO and CH_4_ Oxidation

**DOI:** 10.3390/ma16124257

**Published:** 2023-06-08

**Authors:** Olga Stonkus, Lidiya Kibis, Elena Slavinskaya, Andrey Zadesenets, Ilia Garkul, Tatyana Kardash, Andrey Stadnichenko, Sergey Korenev, Olga Podyacheva, Andrei Boronin

**Affiliations:** 1 Boreskov Institute of Catalysis, Pr. Lavrentieva 5, 630090 Novosibirsk, Russia; stonkus@catalysis.ru (O.S.);; 2 Nikolaev Institute of Inorganic Chemistry, Pr. Lavrentieva 3, 630090 Novosibirsk, Russia

**Keywords:** carbon nanomaterials, composite catalysts, palladium, ceria, CO oxidation

## Abstract

The application of composite materials as catalysts for the oxidation of CO and other toxic compounds is a promising approach for air purification. In this work, the composites comprising palladium and ceria components supported on multiwall carbon nanotubes, carbon nanofibers and Sibunit were studied in the reactions of CO and CH_4_ oxidation. The instrumental methods showed that the defective sites of carbon nanomaterials (CNMs) successfully stabilize the deposited components in a highly-dispersed state: PdO and CeO_2_ nanoparticles, subnanosized PdO_x_ and Pd_x_Ce_1−x_O_2−δ_ clusters with an amorphous structure, as well as single Pd and Ce atoms, are formed. It was shown that the reactant activation process occurs on palladium species with the participation of oxygen from the ceria lattice. The presence of interblock contacts between PdO and CeO_2_ nanoparticles has an important effect on oxygen transfer, which consequently affects the catalytic activity. The morphological features of the CNMs, as well as the defect structure, have a strong influence on the particle size and mutual stabilization of the deposited PdO and CeO_2_ components. The optimal combination of highly dispersed PdO_x_ and Pd_x_Ce_1−x_O_2−δ_ species, as well as PdO nanoparticles in the CNTs-based catalyst, makes it highly effective in both studied oxidation reactions.

## 1. Introduction

Metal–oxide catalysts are widely used in a variety of heterogeneous catalytic reactions required to purify the atmosphere from harmful pollutants, such as CO and hydrocarbons [1]. This is of importance both to neutralize the hazardous components of gasoline and natural gas vehicle exhausts and to clean indoor air [2,3,4].

Cerium oxide (ceria) serves as an indispensable support for platinum group metals (PGM) in CO and hydrocarbon oxidation catalysts due to its high oxygen capacity and oxygen mobility, and its ability to accumulate and release oxygen under redox conditions [5,6]. The strong interaction between PGM and CeO_2_ provides the ability to stabilize deposited PGMs in a highly dispersed state. Thus, due to the synergism between the components, PGM and ceria, the high activity of Pd/CeO_2_ and Pt/CeO_2_ catalysts in CO oxidation at low temperatures can be achieved [7,8]. Active CO oxidation catalysts comprise metal ionic forms embedded in the fluorite structure of the CeO_2_ support, as well as subnanosized clusters/species attached to its surface [8,9,10]. Among PGM/CeO_2_ catalysts, palladium-based catalysts show the highest activity in the methane oxidation reaction [11,12]. To provide the activity of these catalysts in methane oxidation, it is important to maintain the active palladium species over a wide temperature range. The deactivation of Pd/CeO_2_ catalysts occurring at elevated temperatures is associated with a decrease in dispersion due to the sintering of active particles [11,13,14]. To improve the stability of methane oxidation catalysts, the authors of [15] managed to achieve the stability of the active component through the fabrication of core-shell structures, fixing PdO inside the CeO_2_ shell. The authors of [16] used the one-step dry ball-milling method, which also allowed them to synthesize core-shell structures, in which the shell contains Pd embedded in an amorphous ceria layer while the core consists of CeO_2_. In this catalyst, the active components are PdO_x_ species embedded into the CeO_2_ surface layer, the strong interaction between which ensures an easy redox exchange between Pd and CeO_2_. The comprehensive review [12] discusses that by forming a certain combination of Pd in different oxidation states (Pd^0^, Pd^2+^, Pd^4+^, sub-stoichiometric PdOx, etc.), higher catalyst activity can be achieved compared to PdO-based systems. The possibility to stabilize certain forms of palladium on the support surface and their stability under the conditions of oxidative reactions are determined by the metal–support interaction, which means that the structure and, in particular, the defective structure of the support play a key role.

The fabrication of a defective highly dispersed CeO_2_-based support is the key to creating highly active oxidation catalysts. The pure Pd/CeO_2_ system does not possess sufficient thermal stability, and for use at elevated temperatures, CeO_2_ is doped with transition metal ions [17]. Moreover, the fixation of components on the surface of a thermostable support, such as Al_2_O_3_, is widely used, allowing for the stabilization of the dispersed deposited components [18,19].

Carbon materials are widely used in catalysis both independently as catalysts and as supports of heterogeneous catalysts [20,21]. The rather high surface area of carbon materials and their tunable porosity allow for the efficient dispersion of metals and oxides on their surface, thus forming supported metal–carbon catalysts [22], as well as more complex multi-component composite catalysts [23,24,25].

In our previous work [23], we tested carbon nanotubes as a support to stabilize Pd or Pt and CeO_2_ in the dispersed state. We supposed that a carbon support could increase catalysts’ stability in a humid atmosphere, which is important for actual catalyst applications [26]. Of particular interest is the use of various nanostructured graphene-based carbon materials, namely different carbon nanofibers that differ in the packaging of the graphene layers (herring-bone or deck of cards). Of special note are carbon nanotubes, in the structure of which the graphene layers are arranged almost parallel to the growth axis of tube. Various nanostructured carbon materials can differ significantly in their structural characteristics and physicochemical surface properties. Consequently, the nature and structure of the carbon support can have a significant influence on the catalytic properties of the samples. Defects in carbon materials, including point, and extended defects with the presence of various functional groups serve as the centers of stabilization of the highly dispersed supported components up to the single-atom state [27]. Moreover, the dispersion and the nature of the supported components on the carbon surface are determined by the morphology, the structure of the carbon material, the porous structure and the hydrophobic/hydrophilic properties of the surface. The hydrophobic/hydrophilic properties of carbon materials affects the deposition of the active component, but the surface chemistry of carbon materials can easily be modified, for example, by oxidation, to increase their hydrophilicity and favor ionic exchange [20].

For application in catalytic reactions, it is preferable to use carbon materials with a high surface area and a pronounced mesoporous structure. The absence of microporosity in nanostructured carbon materials eliminates diffusion and intraparticle mass transfer in the reaction medium [20].

The main catalytic characteristics of composite catalysts are primarily determined by the chemical state of the active Pd/CeO_2_ components. However, the role of the support in the dispersion and local distribution of the active components, as well as the interaction between the active phase and the support, are also important [28]. The aim of this work is to study the structural, physical and chemical properties of the composite catalysts Pd-CeO_2_/CNMs. We used highly ordered carbon nanofibers and nanotubes as well as disordered carbon material of the Sibunit family. The latter material is of great interest for use in heterogeneous catalytic reactions due to its high resistance to attrition and mechanical crushing, as well as its chemical inertness [29]. All CNMs are characterized by a developed mesoporosity in the absence of micropores. For comparison, the conventional Pd-CeO_2_/Al_2_O_3_ was also synthesized and studied. The catalysts were tested in CO and CH_4_ oxidation reactions. These industrially important reactions are also widely used for testing the active surface centers. The catalysts were characterized by a complex of physicochemical methods, including XRD, TEM and XPS.

## 2. Materials and Methods

### 2.1. Synthesis

Carbon nanotubes (CNTs) and nanofibers (CNFs) were synthesized by the decomposition of 100% ethylene [30]. The catalysts of 62%Fe-8%Ni-30%Al_2_O_3_ and 65%Ni-25%Cu-10%Al_2_O_3_ composition were used as the growth catalysts for CNTs and CNFs, respectively. The synthesized CNTs and CNFs were washed from the growth catalyst by boiling in 2 M HCl for 6 h. The samples were then washed of chlorine ions by repeated boiling in distilled water until the negative reaction when AgNO_3_ was added to the washed water. The washed samples were dried in an Ar flow at 170 °C for 2 h, and then stored in sealed containers. Sibunit mesoporous material was chosen as a commercial carbon support. A broad class of commercial carbon materials of the Sibunit family includes carbon black and pyrolytic carbon composites, which are produced by the decomposition of natural gas on carbon black grains with subsequent activation, leading to the partial or complete removal of carbon black particles and the formation of a porous structure [31]. A carbon support of the Sibunit family (batch no. 4) was provided by the developer’s company—Center of New Chemical Technologies of the Boreskov Institute of Catalysis (Omsk affiliated department), Russia.

Pd-CeO_2_/CNMs catalysts, as well as CeO_2_/CNTs comparison support, were obtained using a previously developed method of co-impregnation of CNMs with active component precursors from acetone solutions [25]. Pd(NO_3_)_2_·2H_2_O palladium nitrate (synthesized following the procedure described by Khranenko et al. [32]) and ammonium cerium(IV) nitrate (NH_4_)_2_[Ce(NO_3_)_6_] (CAN, Rare metals plant, Novosibirsk, Russia) were used as precursors. The weight of the precursors was calculated to achieve the desired composition in the resulting samples, 6 wt.% Pd and 20 wt.% CeO_2_. The reagents were dissolved in acetone and added to the CNMs. The mixture was treated with ultrasound for 10 min and kept in a closed container for 12 h. The acetone was evaporated for 12 h in air and then for 24 h in a desiccator at a pressure of *p*~0.1 atm. The synthesized samples were heated in He to 200 °C at a rate of 10 °C/min, then to 350 °C at a rate of 1 °C/min and kept for 30 min at 350 °C, followed by cooling to room temperature. Further thermal activation involved heating the samples in 1% O_2_ to 350 °C in the reactor and keeping at this temperature for 2 h.

The Pd-CeO_2_/Al_2_O_3_ sample was prepared by the incipient wetness impregnation method. For 0.50 g Al_2_O_3_, 0.40 g CAN and 0.100 g Pd(NO_3_)_2_·2H_2_O were taken. The cerium and palladium precursors were dissolved in 3 mL of acetone. The impregnation was performed in several iterations with an intermediate rapid drying of the support in a flow of warm air. The impregnated sample was calcined similarly as described above. Further thermal activation involved heating the Pd-CeO_2_/Al_2_O_3_ sample in 1% O_2_ to 450 °C in the reactor and keeping at 450 °C for 2 h.

The catalysts synthesized were designated as the Pd-CeO_2_/support, where “support” denotes the carbon or oxide support: CNTs, CNFs, Sibunit or Al_2_O_3_. The text also uses the designation Pd-CeO_2_/CNMs to summarize all the catalysts deposited on various carbon nanomaterials. The reference support, which does not contain palladium, was designated as CeO_2_/CNTs.

### 2.2. Samples Characterization

#### 2.2.1. X-ray Diffraction Analysis (XRD)

X-ray diffraction patterns were obtained on a STOE STADI MP diffractometer (STOE, Darmstadt, Germany) using MoK_α1_ radiation (λ = 0.70926 Å). To generate the primary beam, a curved Ge(111) monochromator was used. The analysis was carried out in the transmission mode. A DECTRIS MYTHEN multichannel detector was used to record the signal. The scanning was performed in the 2Θ range of 5–40° with a step of 0.0015° and an accumulation time of 10 s.

The phase analysis was performed using the ICDD PDF-2 (2009) powder database. Full-profile modeling by the Rietveld method to refine the cerium oxide lattice parameter as well as to estimate the size of the coherent scattering regions (CSR) and the lattice microstrain parameter was performed in the Topas v4.2 program (Bruker, Germany). An external standard, LaB_6_, analysed under similar conditions, was used to account for the instrumental broadening.

#### 2.2.2. X-ray Photoelectron Spectroscopy (XPS)

XPS spectra were obtained on an ES300 (KRATOS Analytical, Manchester, U.K.) using MgKα X-rays (hν = 1253.6 eV). The calibration of the spectrometer energy scale was performed using the spectra of internal levels of gold (Au4f_7/2_) and copper (Cu2p_3/2_), with binding energies (Е_b_) of their peaks taken equal to 84.0 eV and 932.7 eV, respectively. The quantitative surface composition was estimated from the spectral area of the internal levels, taking into account the atomic sensitivity factors (ASF) for the corresponding elements [33]. The Pd3d and Ce3d spectra were decomposed into individual components using a combination of Gauss and Laurentz functions. The scattered electrons background was substracted by the Shirley method. The Ce3d spectra were decomposed into individual components related to the Ce^3+^ states (v^0^, v′ and u^0^, u′) and to the characteristics of the Ce^4+^ ions (v, v″, v‴ and u, u″, u‴) [34]. The percentage of Ce^3+^ ions was determined as the ratio of the area of the v^0^, v′ and u^0^, u′ components to the total Ce3d spectrum area.

#### 2.2.3. Transmission Electron Microscopy (TEM)

The samples were studied by transmission electron microscopy (TEM) using a double aberration-corrected Themis Z electron microscope (Thermo Fisher Scientific, Waltham, MA, USA) operated at an accelerating voltage of 200 kV. Dark-field images were obtained in the scanning (STEM) mode using a HAADF (High-Angle Annular Dark-Field) detector. The instrument is equipped with a Super-X (Thermo Fisher Scientific) EDX detector to perform local elemental analysis of the samples. The crystal lattices on the obtained (S)TEM images were analyzed using the Fourier method. Copper grids (d = 3 mm) covered with a holey carbon film were used as a substrate for the samples. Alcohol suspensions of the samples were dispersed by ultrasound at a frequency of 35 kHz and deposited on the substrates.

### 2.3. Catalytic Properties

#### 2.3.1. Catalytic Activity Measurements

The catalytic properties of the samples in the CO and CH_4_ oxidation reactions were tested using a flow reactor in the temperature-programmed reaction mode (TPR-CO+O_2_ and TPR-CH_4_+O_2_). A RGA 200 (SRS) quadrupole mass spectrometer was used to analyze the gas mixture. The weight and grain size of the Pd-CeO_2_/CNMs catalysts were 0.1 g and 0.25–0.5 mm, respectively. For the Pd-CeO_2_/Al_2_O_3_ catalyst, the weight and grain size were 0.1 g and 0.14–0.25 mm, respectively.

The initial reaction mixture contained 0.2 vol% CO, 1.0 vol% O_2_, 0.5 vol% Ne and He as a balance gas for the CO oxidation reaction. The flow rate was 1000 cm^3^/min, which corresponds to a GHSV of 600 L × g^−1^ × h^−1^. For the CH_4_ oxidation reaction, the initial reaction mixture contained 0.1 vol% CH_4_, 1.0 vol% O_2_, 0.5 vol% Ne and He as a balance gas. The flow rate was 100 cm^3^/min, which corresponds to a GHSV of 60 L × g^−1^ × h^−1^. The reaction mixture was admitted to the reactor at −40 °C or 50 °C for CO and CH_4_ oxidation reactions, respectively. After the steady-state concentrations of the reagents were established, the catalyst was heated at a heating rate of 10 °C/min to 350 °C. The experiments included three consecutive heating cycles with intermediate cooling in the reaction mixture.

#### 2.3.2. Temperature Programmed Reduction

The TPR-CO curves were obtained using the reaction mixture containing 1.0 vol.% CO, 0.5 vol.% Ne and He. The gas mixture was introduced at a flow rate of 100 cm^3^/min to the catalyst sample (0.1 g), pre-cooled in the reactor to −40 °C. As the steady-state concentrations of CO and CO_2_ were established, the sample was heated to 450 °C at a 10 °C/min heating rate. The concentrations of CO, CO_2_, O_2_, H_2_ and H_2_O were monitored during the reaction by a mass-spectrometer at a frequency of 0.27 Hz. Before each TPR-CO experiment, the catalysts were pre-treated with a 1%O_2_/He gas mixture at 350 °C for 2 h, with subsequent cooling in this mixture and purging in helium afterwards.

## 3. Results and Discussion

### 3.1. Textural Properties and XRD Data

The textural characteristics of the used supports are provided in Table 1.

**Figure 1:**
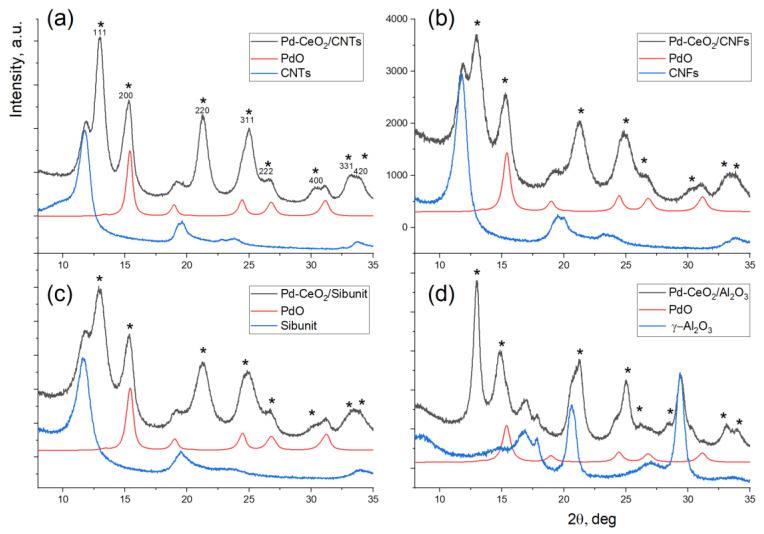
X-ray diffraction patterns of Pd-CeO_2_/CNTs (**a**), Pd-CeO_2_/CNFs (**b**), Pd-CeO_2_/Sibunit (**c**) and Pd-CeO_2_/Al_2_O_3_ (**d**) shown as black curves. X-ray diffraction patterns of the corresponding supports are shown in blue; the scattering contribution from the PdO phase is shown in red. The *-sign indicates the reflexes of the CeO_2_ phase.

Figure 1 shows the X-ray diffraction patterns of the catalysts and of the corresponding supports. The main phases detected in the Pd-CeO_2_/CNMs catalysts are cerium oxide CeO_2_ with a fluorite-type structure (ICDD PDF-2 #00-034-0394), and a carbon material phase whose diffraction pattern does not vary much for different types of supports. In addition, a highly dispersed palladium oxide PdO phase (ICDD PDF-2 #00-41-1107) is detected on all XRD patterns.

To calculate and refine the structural and microstructural parameters of the detected phases, a full-profile modeling of the experimental diffraction data was carried out. The diffraction pattern of the carbon support or aluminum oxide was described by a set of individual peaks. The positions, widths and ratios of the intensities between these peaks were refined relative to the diffraction pattern of the pure support. The obtained parameters of individual diffraction maxima were then fixed and used to describe the diffraction pattern of the catalysts. The scattering from cerium oxide and palladium oxide was modeled using data on the crystal structure of these phases. In the case of the Pd-CeO_2_/Al_2_O_3_ sample, the precise calculation of the CSR size for the PdO phase was rather complicated due to the strong overlap of its lines with the lines of the Al_2_O_3_ support. The refined parameters of the cerium oxide and palladium oxide phases are provided in Table 2.

It was found that CeO_2_ and PdO particles in the composite catalysts are characterized by a small CSR size: 2–7 nm for cerium oxide, and 3–12 nm for palladium oxide. In addition, sufficiently large values of the microstrain parameter (e_0_) were obtained for these phases. This parameter describes the standard deviation of the interplanar distances from the average value. A high value of the microstrain parameter (above 0.1) is characteristic of defective structures. It can be noted that the highest CSR values for CeO_2_ were obtained for the catalyst deposited on Al_2_O_3_, and the highest CSR value for the PdO phase was obtained for the catalyst deposited on CNTs. An estimation of the phase content from the X-ray diffraction patterns for carbon-based catalysts showed that about 80% of the introduced palladium is in the form of PdO.

### 3.2. TEM Data

**Figure 2:**
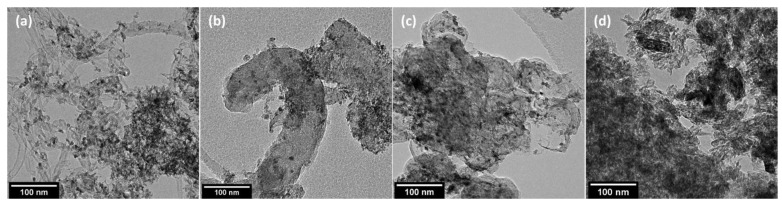
TEM images of (**a**) Pd-CeO_2_/CNTs, (**b**) Pd-CeO_2_/CNFs, (**c**) Pd-CeO_2_/Sibunit and (**d**) Pd-CeO_2_/Al_2_O_3_. The images are presented at the same magnification.

According to TEM data, CNTs have a diameter of 8–20 nm and the tube walls are composed of 6–20 graphene layers (Figure 2a). The CNFs with diameters ranging from 10 to 100 nm have a herring-bone structure (Figure 2b). The Sibunit support comprises the hollow carbon fragments of a spherical or near-spherical shape, ranging in size from 50 to 200 nm (Figure 2c). Aluminum oxide is represented by agglomerates of needle-shaped crystals with sizes d = 4–8 nm and l = 15–50 nm, and the size of agglomerates is 50–200 nm.

TEM images definitely show the presence of both single deposited nanoparticles and the agglomerates of nanoparticles of different sizes on the surface of all carbon and Al_2_O_3_ supports. Nevertheless, the morphology and nature of the support affect the agglomeration and distribution features of the deposited components.

**Figure 3:**
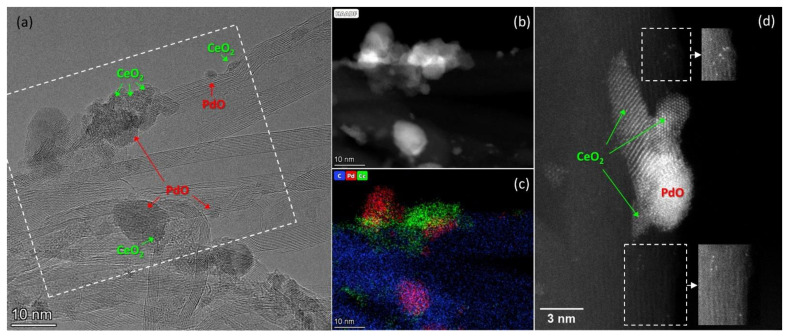
TEM data for Pd-CeO_2_/CNTs catalyst: (**a**) HRTEM image; (**b**,**c**) HAADF-STEM image of the region marked in figure (**a**), and a corresponding EDX-mapping pattern showing the distribution of carbon (blue), palladium (red) and cerium (green) in the selected region; (**d**) HAADF-STEM image showing an agglomerate of PdO and CeO_2_ nanoparticles on the CNTs’ surface. The insets in figure d show the images of selected areas with brightness/contrast correction for better visualization of single Pd and Ce atoms present on the surface of CNTs.

For example, when palladium and cerium oxide are deposited on CNTs, both individual nanoparticles of PdO and CeO_2_ and agglomerates of deposited particles up to 100 nm in size are formed. The extended sections of the tubes contain a small number of supported particles. High-resolution TEM images show that the stabilization of single nanoparticles and agglomerates having a size of about 10–30 nm occurs predominantly near the surface defects of the tubes, such as breaks in the surface graphene layers, as well as in regions with various bends of the tubes (Figure 3a). Earlier, in [23] we observed that nanoparticles can also be found in the inner cavity of the tubes, although the number of such particles visible on TEM images is usually small. Figure 3a–c show HTREM and HAADF-STEM images of the same sample region and the corresponding EDX mapping patterns. It can be seen that the PdO nanoparticles in the agglomerates are about 8–10 nm in size and are somewhat larger than the contacting CeO_2_ nanoparticles. However, the single PdO nanoparticles located on the surface of CNTs and not contacting with CeO_2_ have a smaller size of about 2 nm (Figure 3). The size of CeO_2_ particles also varies depending on their location, and is about 2 nm for single crystallites contacting with CNTs only, and reaches 3–7 nm for crystallites comprising PdO-CeO_2_ agglomerates. The HAADF-STEM study also revealed the presence of single atoms on the surface of CNTs (Figure 3d). The non-uniformity of the support contrast does not allow us to reliably attribute the bright points observed on the images to Pd or Ce atoms, but the difference in brightness of the two closest dots in the same region of the CNTs suggests that both types of single atoms are present on the surface. Previously, in a study of catalysts with a single active component (Pd/CNTs or CeO_2_/CNTs), we showed that both Pd and Ce can be stabilized as single atoms on the surface of CNTs [26].

**Figure 4:**
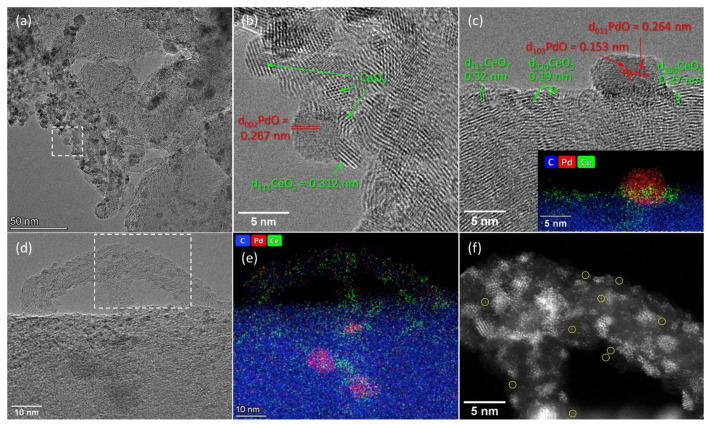
TEM data for Pd-CeO_2_/CNFs catalyst: (**a**) TEM image; (**b**) HRTEM image of the region marked in figure (**a**); (**c**) HRTEM image and an EDX-mapping pattern (in inset) of the same region; (**d**–**f**) TEM image, corresponding EDX-mapping pattern and a HAADF-STEM image of the marked region. The following colors are used for the EDX elemental maps: blue for carbon, red for palladium and green for cerium. The maps are presented in background-corrected intensities. The yellow circles indicate single Pd and Ce atoms on the CNFs surface.

Carbon fibers as a support also provide the effective dispersion of the deposited nanoparticles over the surface. However, the density of nanoparticles on the surface of different fibers varies. This is apparently due to the wide variation in the diameter of the fibers and the heterogeneity of their surface defects. Consequently, the agglomerates of nanoparticles are formed around some fibers, tightly contacting each other and adhering to the fiber surface (Figure 4a), while on the surfaces of other tubes, single nanoparticles are formed (Figure 4d). The inner cavity of the carbon fibers is small, and the bridges of the graphene layers crossing this cavity are visible. Thus, the inner surface of the fibers is inaccessible to the deposited components, so the deposited components in this specimen are located only on the outer fiber surface. The size of the PdO particles observed in the TEM images is 5–8 nm. The size of the CeO_2_ nanoparticles is 1–5 nm, and larger particles are observed in dense agglomerates. Interestingly, no isolated PdO nanoparticles could be detected in this sample by TEM. As can be seen from Figure 4b,c, the PdO nanoparticles are surrounded and in tight contact with the CeO_2_ nanocrystallites. Figure 4d–f show a sample region containing a carbon nanofiber with a diameter of 10 nm, on the surface of which multiple CeO_2_ nanoparticles with the size of 1–2 nm are located. The HAADF-STEM image (Figure 4f) also shows the presence of disordered subnanosized clusters and single atoms on the surface. EDX data show the presence of both Pd and Ce signals in this region, with an atomic ratio of Ce:Pd ≈ 3:2. Since palladium can be incorporated into the fluorite structure of CeO_2_ [35,36], we assume the formation of highly dispersed and highly defective Pd_x_Ce_1−x_O_2−δ_ species in the form of nanoparticles (1–2 nm) with a fluorite structure and amorphous subnanosized clusters in such areas of the sample.

**Figure 5:**
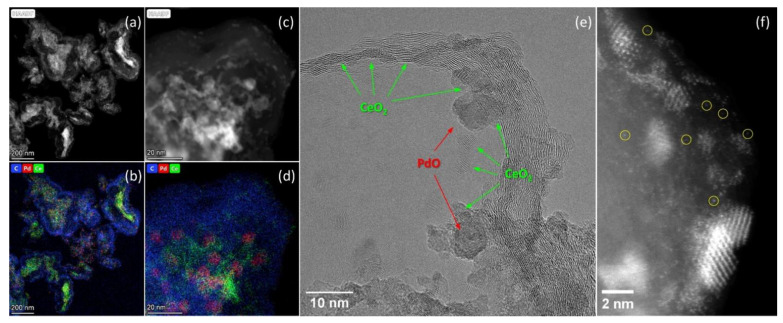
TEM data for Pd-CeO_2_/Sibunit: (**a**–**d**) HAADF-STEM images and corresponding EDX-mapping patterns; (**e**) I HRTEM image; (**f**) HAADF-STEM image, the yellow circles indicate single Pd and Ce atoms on Sibunit surface. The EDX elemental maps use the following colors: blue for carbon, red for palladium and green for cerium. The maps are presented in background-corrected intensities.

In the Sibunit-based catalyst, the morphology of the support has a significant influence on the distribution of the agglomerates of PdO-CeO_2_ nanoparticles. The HAADF-STEM images and the corresponding EDX mapping patterns (Figure 5a–d) show that the agglomerates of deposited particles are located preferentially in the inner cavities of the Sibunit “globules”. Since the “globules” have open sections, the components in the inner cavity remain accessible to the reaction medium, but the activity of the catalyst can be limited by diffusion. The agglomerates in the inner cavities of the Sibunit contain PdO nanoparticles with a size of approximately 5–7 nm and CeO_2_ nanoparticles with a size of about 2–4 nm (Figure 5c–e). The PdO nanoparticles are in contact with the carbon support and CeO_2_ nanoparticles, but almost do not contact each other.

The surface of the Sibunit is represented by curved graphene sheets, which can come to the surface at different angles, especially in the areas where the wall thickness changes, and at the open edges of the “globules” (Figure 5e). Thus, the surface structure of the Sibunit combines areas similar to the surface of CNTs (extended graphene sheets) and the surface of CNFs (inclined graphene sheets). The latter, due to their defectiveness, are more preferable for fixing single nanoparticles, which determines some heterogeneity in the distribution of the deposited components over the outer surface of the support in the Pd-CeO_2_/Sibunit catalyst. Similar to the Pd-CeO_2_/CNFs catalyst, the Sibunit surface contains both single deposited atoms and Pd_x_Ce_1−x_O_2−δ_ clusters or nanoparticles with an amorphous or fluorite structure (Figure 5f).

Therefore, according to TEM data, the following forms of the deposited components in Pd-CeO_2_/CNMs composite catalysts can be distinguished: (1) the deposited components anchored on the carbon surface in the form of single nanoparticles (PdO or CeO_2_) not interacting with each other; (2) agglomerates of PdO and CeO_2_ nanoparticles of different sizes, in which interblock boundaries between PdO and CeO_2_ crystallites are formed; (3) subnanosized PdO_x_ or Pd_x_Ce_1−x_O_2−δ_ clusters, characterized by an amorphous structure due to their small size, and fixed on the carbon support surface; and (4) Pd and Ce single atoms stabilized on the carbon surface, or embedded in the structure of oxide phases (Pd can be incorporated into the fluorite structure of CeO_2_).

The defectiveness of the carbon surface determines the number of deposited species that can be stabilized in atomically dispersed forms. In the case of the less defective CNTs, the number of single Pd and Ce atoms is visually low, while the defective surface of carbon nanofibers and the Sibunit contains multiple single atoms and subnanosized clusters with a disordered structure. It should also be noted that, for nanostructured carbon materials, the inner and outer surfaces on which the supported component can be fixed are often distinguished [37,38]. If the deposited component is located on the inner surface of the carbon material, which occurs in the case of the Sibunit, and is also possible for CNTs, the access of the reagents to it may be restricted, which can affect the catalytic activity. 

**Figure A1:**
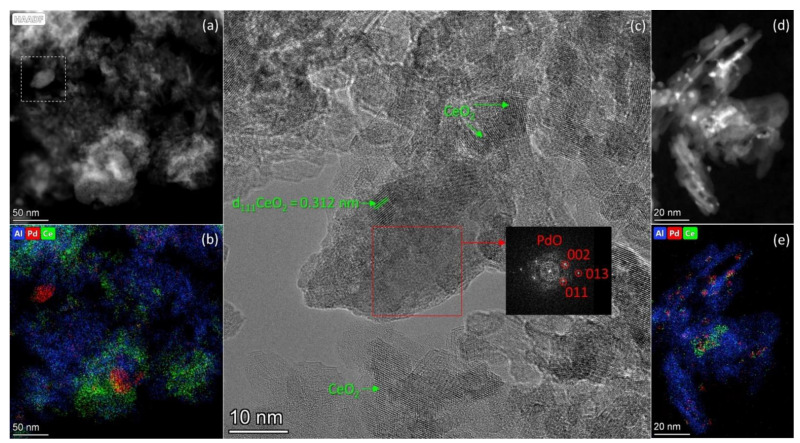
TEM data for Pd-CeO_2_/Al_2_O_3_: (**a**,**b**) HAADF-STEM image and corresponding EDX-mapping pattern; (**c**) HRTEM image with FTT of the selected region shown in the inset; the interplanar distances corresponding to PdO and CeO_2_ are indicated; (**d**,**e**) HAADF-STEM image and corresponding EDX-mapping pattern. The square mark in the figure (**a**) indicates the region of the sample shown in the HRTEM image (**c**). The EDX elemental maps use the following colors: blue for Al, red for Pd and green for Ce. The maps are presented in background-corrected intensities.

The TEM data for the Pd-CeO_2_/Al_2_O_3_ reference catalyst are shown in Figure A1. This catalyst is characterized by a non-uniform distribution of the deposited components over the Al_2_O_3_ support. A part of the deposited component forms agglomerates up to 100 nm in size (Figure A1a,b), weakly interacting with the Al_2_O_3_ support, while another part is fixed on Al_2_O_3_ in the form of small agglomerates and single nanoparticles (Figure A1d,e). CeO_2_ nanoparticles in the agglomerates have a size of 3–10 nm and are located both on the Al_2_O_3_ support and on the surface of larger PdO nanoparticles. The size of PdO particles located on the Al_2_O_3_ support in the absence of contact with CeO_2_ is about 2 nm. Larger, 10–20 nm, single PdO nanoparticles are present in some areas of the sample. Large PdO particles are characterized by the presence of contacts with CeO_2_ crystallites (Figure A1a–c).

Thus, in Pd-CeO_2_/CNMs and Pd-CeO_2_/Al_2_O_3_ composite catalysts, the stabilization of various forms of the deposited component is determined by the structure and defectiveness of the support surface. The nanoparticles and clusters of the active components are anchored as separate species on the local defects of the support surface, which provides their spatial distribution without agglomeration. The formation of single atoms also increases the dispersion of the deposited components. This is more characteristic of CNFs and Sibunit supports due to the stronger interaction of Pd and Ce species with the carbon surface. In addition, the structure and morphology of the carbon materials influence the agglomeration of PdO and CeO_2_ nanoparticles. The nanoparticles comprising the agglomerates are larger than the single ones stabilized on the support.

### 3.3. XPS Data

Table 3 summarizes the surface composition data obtained by XPS for Pd-CeO_2_/CNMs and Pd-CeO_2_/Al_2_O_3_ catalysts. Analysis of the data shows that the concentrations of Pd and Ce in the samples deposited on different carbon nanomaterials do not differ significantly. In the case of CNFs and the Sibunit, the amount of palladium and cerium on the surface is somewhat higher. According to XRD and TEM data, the crystallite size of PdO and CeO_2_ is smaller when deposited on the CNFs and Sibunit supports, which leads to a higher intensity of palladium and cerium lines in the XPS spectra. For the Pd-CeO_2_/Al_2_O_3_ catalyst, the amount of palladium and cerium observed in the XPS spectra is about two times lower than in the case of CNMs-based samples. This fact is obviously related to the stabilization of larger PdO and CeO_2_ particles on the aluminum oxide surface compared to CNMs.

**Figure 6:**
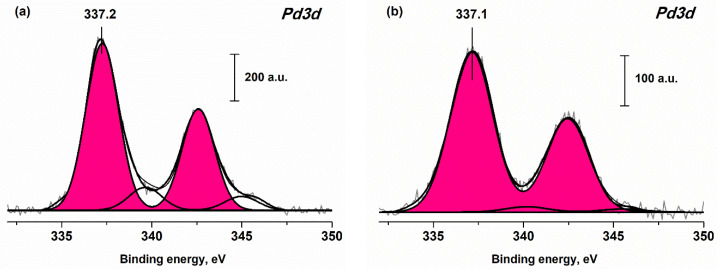
The Pd3d spectra of (**a**) Pd-CeO_2_/CNTs and (**b**) Pd-CeO_2_/Al_2_O_3_ samples.

**Figure A2:**
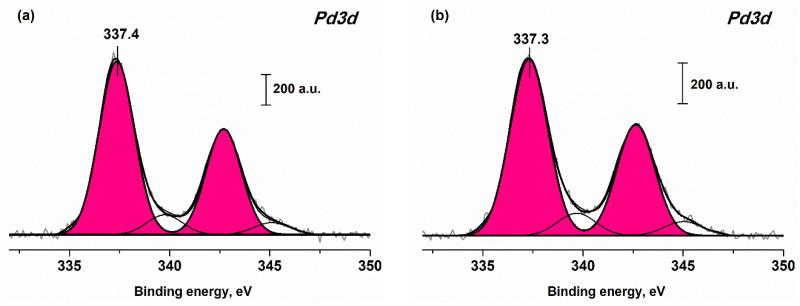
The Pd3d spectra of (**a**) Pd-CeO_2_/CNFs and (**b**) Pd-CeO_2_/Sibunit samples.

Figure 6 presents the Pd3d spectra for Pd-CeO_2_/CNTs and Pd-CeO_2_/Al_2_O_3_ samples. The Pd3d spectra for Pd-CeO_2_/CNFs and Pd-CeO_2_/Sibunit catalysts are presented in Figure A2. The Pd3d spectra for all studied catalysts can be described by a single spin-orbit Pd3d_5/2_-Pd3d_3/2_ doublet with an E_b_(Pd3d_5/2_) value of about 337.1–337.4 eV. A small doublet with E_b_(Pd3d_5/2_)~340 eV can be attributed to the PdO satellite structure [39,40]. The value E_b_(Pd3d_5/2_) = 337.1–337.4 eV is typical of the palladium in a 2+ oxidation state. For palladium oxide PdO, the binding energy is usually observed in the region of 336.6–336.9 eV [39,40,41]. For Pd^2+^ ionic species stabilized in the CeO_2_ matrix, the E_b_ value is about 338.0 eV [36,42,43]. According to structural investigation and TPR-CO data, which will be discussed below, highly dispersed forms of Pd^2+^ and PdO particles of various sizes are observed in the studied catalysts. Apparently, the presence of several palladium states with close binding energy values does not allow us to reliably distinguish them in the Pd3d spectra. However, based on the position of the maximum of the Pd3d_5/2_ peak, we can conclude that among the studied samples, the fraction of palladium oxide PdO is maximal on the surface of the Pd-CeO_2_/Al_2_O_3_ catalyst and minimal in the case of the Pd-CeO_2_/CNFs sample.

Analysis of Ce3d spectra shows that the fraction of Ce^3+^ in the samples based on carbon nanomaterials is close to 20%. For the Pd-CeO_2_/Al_2_O_3_ sample, the Ce^3+^ fraction is slightly lower—15%. The Ce^3+^ content above 15% indicates the formation of defective CeO_2_ particles, with an increased concentration of oxygen vacancies [23,26]. This is consistent with the XRD data indicating the formation of small CeO_2_ nanocrystallites with a high value of the microstrain parameter. The lattice microdistortions induce the formation of Ce^3+^ ions and oxygen vacancies.

### 3.4. TPR-CO+O_2_, TPR-CH_4_+O_2_: Catalytic Data

**Figure 7:**
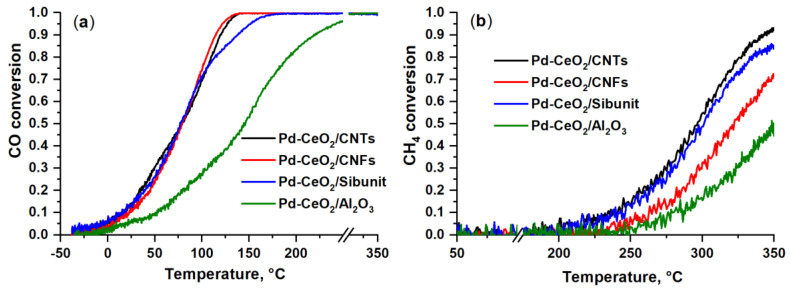
Temperature dependencies of (**a**) CO and (**b**) CH_4_ conversion for Pd-CeO_2_/CNTs, Pd-CeO_2_/CNFs, Pd-CeO_2_/Sibunit and Pd-CeO_2_/Al_2_O_3_ catalysts.

The influence of the support nature was studied for Pd-CeO_2_/CNMs and Pd-CeO_2_/ Al_2_O_3_ catalysts in the CO and CH_4_ oxidation reactions. Figure 7 shows the temperature dependencies of CO and CH_4_ conversion. Table 4 summarizes catalytic data on the behavior of the studied catalysts in CO and CH_4_ oxidation reactions, indicating reference temperatures at which conversions of 10% (T_10_), 50% (T_50_) and 90% (T_90_) are achieved. 

The catalytic study showed that the CNMs-based catalysts are active in the CO oxidation reaction at temperatures below 0 °С and are characterized by a close low-temperature activity. The Pd-CeO_2_/Al_2_O_3_ catalyst manifests activity only at temperatures above 25 °C. In the mid-temperature region, the T_50_ values are close to 78 °C for all CNMs-based samples. At temperatures above 80 °C, the CO conversion dependencies are different. Pd-CeO_2_/CNTs and Pd-CeO_2_/CNFs catalysts are characterized by lower temperatures of achieving a high CO conversion, and T_90_ for these catalysts is 115 and 121 °С, which is about 20 °С lower than for the Pd-CeO_2_/Sibunit catalyst (138 °С). The CO conversion curve for the Pd-CeO_2_/Al_2_O_3_ catalyst is shifted by 70 °С to higher temperatures, relative to the curves for CNMs-based catalysts.

In the case of the CH_4_ oxidation reaction, the lowest temperature to achieve 10% conversion of CH_4_ (T_10_) is observed for the Pd-CeO_2_/CNTs (235 °C) and Pd-CeO_2_/Sibunit (235 °C) catalysts. The methane conversion curves for the Pd-CeO_2_/CNFs and Pd-CeO_2_/Al_2_O_3_ catalysts are shifted toward higher temperatures.

To investigate the activity of the catalyst oxygen with respect to CO, and to identify the forms of oxidized Pd on the catalyst surface, the temperature-programmed reaction with CO in the absence of oxygen in the gas phase (TPR-CO method) was applied.

### 3.5. TPR-CO Data

**Figure 8:**
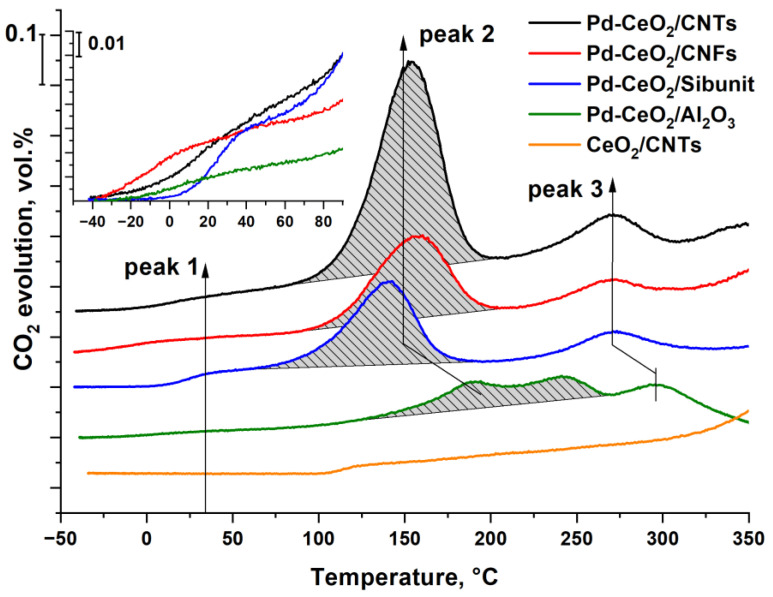
CO_2_ evolution during TPR-CO for Pd-CeO_2_/CNTs, Pd-CeO_2_/CNFs, Pd-CeO_2_/Sibunit, Pd-CeO_2_/Al_2_O_3_ catalysts and for CeO_2_/CNTs sample. The inset shows the initial section of CO_2_ evolution curves in the temperature range of −40–100 °C.

Figure 8 shows the temperature dependencies of CO_2_ evolution during the interaction of the catalysts with CO. The evolution of CO_2_ for the CeO_2_/CNTs reference support starts around 100 °C, and no peaks are observed in the range of 100–300 °C. In agreement with our previous results [44]_,_ CO_2_ evolution occurs at the temperature range of 100–250 °C due to the small activation energy of the interaction of CO and oxygen on the surface of CeO_2_, while the evolution of CO_2_ at temperatures above 300 °C is due to the interaction of CO with the bulk oxygen of CeO_2_ [45,46]. For Pd-containing catalysts, several peaks of CO_2_ evolution are observed in the temperature range up to 300 °C, which allows us to refer them to different forms of oxidized palladium. According to [9,47,48], the peaks of CO_2_ evolution in the region of −40–70 °C (peak 1), 70–200 °C (peak 2) and 250–300 °C (peak 3) refer to active PdO_x_ surface clusters, PdO nanoparticles and CeO_2_ nanoparticles modified by Pd^2+^ ions, respectively.

According to Figure 8 (see inset), the release of CO_2_ for Pd-CeO_2_/CNFs and Pd-CeO_2_/CNTs catalysts starts already at −40 °C (peak 1), while for Pd-CeO_2_/Al_2_O_3_ and Pd-CeO_2_/Sibunit catalysts, CO_2_ is registered at −20 and 0 °C. This indicates that the Pd-CeO_2_/CNTs and Pd-CeO_2_/CNFs catalysts contain more loosely bound reactive oxygen in the PdO_x_ surface clusters. The second peak for these catalysts, which is responsible for the interaction of CO with PdO, is observed at a close temperature of about 150 °C, while for the Pd-CeO_2_/Sibunit catalyst, the T_max_ of the PdO reduction peak is lower and is about 140 °C. This implies that the Pd-CeO_2_/Sibunit catalyst contains PdO particles with more reactive oxygen, which may indicate a higher dispersity or defectiveness of PdO crystallites in this sample. The T_max_ of the third peak of CO_2_ evolution is almost the same for all CNMs-based catalysts. For the Pd-CeO_2_/Al_2_O_3_ catalyst, a shift of all peaks to the high-temperature region is observed. Moreover, in the temperature range of 100–270 °C, two poorly resolved peaks responsible for the interaction of CO with PdO are observed. The separation of these two peaks is probably related to the presence of two types of PdO nanoparticles, which differ not only in size, but also in the presence or absence of contacts with CeO_2_ nanoparticles, according to TEM data.

Table 5 presents quantitative data on the evolved CO_2_ for the second peak, calculated as the area under the corresponding peak. For peak 1, the amount of CO_2_ released in all the catalysts is close and equals 5–10 μmol/g, and the CO_2_/Pd ratio is in the range of 0.009–0.017, which corresponds to ~1–2% of all the Pd in the catalyst. For peak 2, corresponding to the interaction of CO with PdO, the amounts of evolved CO_2_ differ significantly. The CO_2_-2/Pd ratio exceeds 1 for the Pd-CeO_2_/CNTs catalyst and the total amount of evolved CO_2_ for this catalyst is the highest. According to XRD and TEM data, the Pd-CeO_2_/CNTs catalyst comprises larger and well-crystallized PdO and CeO_2_ nanoparticles that are efficiently mixed in PdO-CeO_2_ agglomerates. In this case, during the TPR-CO reaction, the interblock boundaries between PdO and CeO_2_ crystallites provide additional oxygen transfer and involvement of oxygen from CeO_2_ nanoparticles in the PdO reduction process, leading to an increased amount of CO reacting with the PdO as a second peak. The variation of the maximum temperature of the second peak for different catalysts is most likely related not only to the size of the PdO particles, but also to the modification of the CeO_2_ fluorite lattice by Pd^2+^ ions, which facilitate the mobility of oxygen in the fluorite lattice [9].

**Figure 9:**
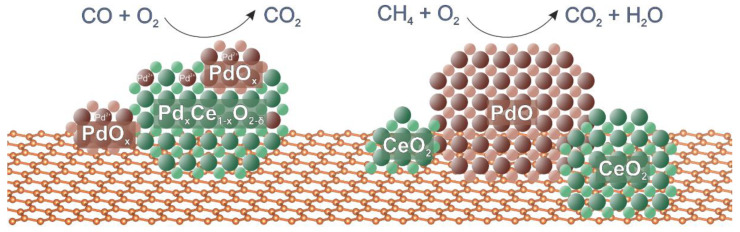
A scheme showing the main types of palladium and ceria species formed on carbon supports: the PdO_x_ clusters and Pd_x_Ce_1−x_O_2−δ_ particles provide activity in low-temperature CO oxidation; the PdO nanoparticles are essential for CH_4_ oxidation.

Thus, the results of the catalytic testing of the studied Pd-CeO_2_/CNMs composites showed that all the samples are characterized by similar catalytic activity in the oxidation of CO. For all carbon-based catalysts, the ignition of the CO oxidation reaction occurs at a low temperature near 0 °C, indicating the high activity of these catalysts. This activity is directly related to the stabilization of highly dispersed PdO_x_ clusters and Pd_x_Ce_1−x_O_2−δ_ particles on the surface of carbon materials’ defects, which can react with CO at around-zero temperatures (Figure 9). The observation of low-temperature TPR-CO peaks at subzero temperatures confirms this assumption.

The notable differences in catalytic activity are observed in CH_4_ oxidation, in which the methane conversion curves have a different temperature shift. CNTs and Sibunit-based catalysts start operating at lower temperatures than the CNFs-based one. The presence of dispersed PdO nanoparticles determines the activity of catalysts in methane oxidation (Figure 9). The possibility of the stabilization of deposited active components in the form of the agglomerates of PdO and CeO_2_ nanoparticles having the contacting boundaries allows for the efficient oxygen transfer from CeO_2_ to PdO. The optimal combination of highly dispersed Pd_x_Ce_1−x_O_2−δ_ species and PdO nanoparticles in the CNTs-based catalyst makes it highly effective in both studied oxidation reactions.

**Figure A3:**
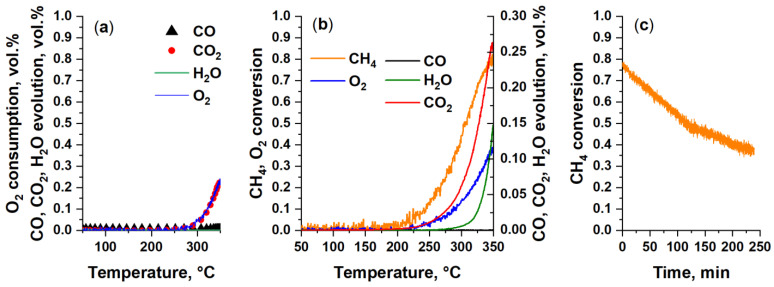
Catalytic data for Pd-CeO_2_/CNTs catalyst: (**a**) Temperature dependence of O_2_ consumption and CO, CO_2_ and H_2_O evolution during temperature-programmed oxidation (TPO) in 1%O_2_/He; (**b**) Temperature dependences of CH_4_, O_2_ conversion and CO, H_2_O and CO_2_ evolution during CH_4_ oxidation; (**c**) Time dependence of CH_4_ conversion at 350 °C.

It should be noted that when the carbon-based samples interact with oxygen, the oxidation of the carbon support with the evolution of CO_2_ at temperatures above 300 °C is possible (Figure A3a). It is important to note that CO is not formed until 350 °C. During the reaction of CH_4_ oxidation, only CO_2_ and H_2_O are observed as reaction products, while CO formation does not occur (Figure A3b). At the same time, the evolution of water is observed at a temperature of 280 °C, which is 100 °C higher than the start temperature of CH_4_ oxidation (180 °C). We associate this with the decomposition of the hydroxide that can form during the interaction of PdO with CH_4_. Isothermal tests for 4 h at 350 °С show the gradual deactivation of the catalyst (Figure A3c) so that the CH_4_ conversion decreases from 77 to 30%. Therefore, further studies are needed to improve the stability of Pd-CeO_2_/CNMs catalysts.

## 4. Conclusions

Highly dispersed composite catalysts containing palladium and CeO_2_ supported on nanostructured carbon materials were synthesized and studied in CO and CH_4_ oxidation reactions. The carbon materials of different structures (CNTs, CNFs, Sibunit) were found to be effective in stabilizing the active forms of the deposited Pd-CeO_2_ composite components. Cerium is stabilized in the form of small CeO_2_ nanoparticles ranging in size from 1 to 5 nm, while palladium is in the Pd^2+^ state, forming subnanosized PdO_x_ clusters and PdO nanoparticles with a size of 2–12 nm. The defective structure of the carbon support surface is responsible for anchoring the highly dispersed nanoparticles and clusters.

Carbon-based catalysts are characterized by close activity in the oxidation of CO, starting to work at around-zero temperatures. The activity in methane oxidation differs, and is determined both by the dispersion of PdO nanoparticles and by the presence of PdO-CeO_2_ interblock contacts, providing oxygen transfer. The data obtained indicate the good accessibility of active palladium species supported on carbons having a different morphology.

The use of aluminum oxide as a reference support leads to the formation of the same forms of the active component, but their distribution over the support surface differs considerably. Dispersed PdO nanoparticles are stabilized on the Al_2_O_3_ surface as separated entities without contact with CeO_2_ particles. This significantly affects the oxygen transfer from the ceria lattice to the reagents adsorbed on palladium species, so the activity of the Pd-CeO_2_/Al_2_O_3_ catalyst in CO and CH_4_ oxidation is lower than that of all Pd-CeO_2_/CNMs systems.

Thus, carbon materials with different structures could be efficiently used as supports in composite catalysts. By using carbon materials with varying structures, defectiveness and morphologies, it is possible to anchor the deposited active components in the desired structural and electronic state.

## Figures and Tables

**Table 1:** Textural characteristics of the CNMs and Al_2_O_3_ supports.

Support	S_BET_, m^2^/g	V_pore_, cm^3^/g	V_micro_, cm^3^/g	D_pore_, nm
CNTs	179	1.29	0	29
CNFs	197	0.45	0	9
Sibunit	313	0.44	0.01	6
Al_2_O_3_	166	0.45	0	11

**Table 2:** Structural and microstructural parameters of phases in Pd-CeO_2_/CNMs and Pd-CeO_2_/Al_2_O_3_ samples.

Sample	CeO_2_ Parameters			PdO Parameters	
	а, Å	D, nm	e_0_	D, nm	e_0_
Pd-CeO_2_/CNTs	5.411(1)	4(1)	0.18(3)	12(2)	0.34(3)
Pd-CeO_2_/CNFs	5.414(3)	2.7(3)	0.16(4)	6(1)	0.1(2)
Pd-CeO_2_/Sibunit	5.40(1)	2.4(3)	0.10(3)	6(1)	0.12(4)
Pd-CeO_2_/Al_2_O_3_	5.41(1)	7(1)	0.3(1)	≈3 *	-

* a precise value is not possible to calculate because of the strong overlap of the phase lines with the aluminum oxide lines.

**Table 3:** XPS data on the quantitative composition of the catalysts’ surface (atomic %); the binding energy of the Pd3d_5/2_ line (E_b_(Pd3d_5/2_)), and the Ce^3+^ fraction calculated based on the Ce3d spectra.

Sample	Pd, at%	Ce, at%	E_b_(Pd3d_5/2_), eV	Се^3+^, %
Pd-CeO_2_/CNTs	0.85	3.5	337.2	19
Pd-CeO_2_/CNFs	1.1	3.8	337.4	20
Pd-CeO_2_/Sibunit	1.0	3.6	337.3	23
Pd-CeO_2_/Al_2_O_3_	0.5	2.8	337.1	15

**Table 4:** Catalytic properties.

Sample	CO Oxidation			CH_4_ Oxidation
	T_10_, °C	T_50_, °C	T_90_, °C	T_10_, °C
Pd-CeO_2_/CNTs	14	78	115	235
Pd-CeO_2_/CNFs	20	78	121	265
Pd-CeO_2_/Sibunit	14	78	138	235
Pd-CeO_2_/Al_2_O_3_	51	145	220	280

**Table 5:** TPR-CO data: T_2_—temperature of peaks associated with the interaction of CO with PdO, T_3_—temperature of peaks associated with the interaction of CO with CeO_2_ modified by Pd^2+^ ions, CO_2_-2 and CO_2_-Σ—the amount of CO_2_ evolved during the second peak and total; CO_2_-2/Pd—the ratio of the amount of CO_2_ evolved during the second peak to the palladium content in the catalyst.

Catalyst	T_2_,°C	T_3_,°C	CO_2_-2, μmol/g	CO_2_-2/Pd	CO_2_-Σ, μmol/g
Pd-CeO_2_/CNTs	152	268	722	1.28	2090
Pd-CeO_2_/CNFs	153	264	322	0.57	1450
Pd-CeO_2_/Sibunit	138	270	315	0.56	1090
Pd-CeO_2_/Al_2_O_3_	190, 225	285	170	0.30	928

## Data Availability

The data presented in this study are available on request from the corresponding author.
